# Flavor profile of “Dao Ban Xiang” (a traditional dry‐cured meat product in Chinese Huizhou cuisine) at different processing stages in winter and summer

**DOI:** 10.1002/fsn3.3225

**Published:** 2023-05-04

**Authors:** Hanlin Zhu, Ping Li, Lin Wang, Qianli Huang, Baocai Xu

**Affiliations:** ^1^ School of Food and Biological Engineering Hefei University of Technology Hefei China; ^2^ Engineering Research Center of Bio‐Process, Ministry of Education Hefei University of Technology Hefei China

**Keywords:** Dao Ban Xiang, free amino acids, free fatty acids, physicochemical properties, volatile flavor compounds

## Abstract

“Dao Ban Xiang” is a famous traditional Chinese dry‐cured meat product. This study aimed to comparatively analyze the difference in the volatile flavor information of “Dao Ban Xiang” produced in winter and summer. In this study, we determine the physical and chemical properties, free amino acids (FAAs), free fatty acids (FFAs), and volatile compounds in the four processing stages of samples in winter and summer. The content of FAAs decreased significantly during the curing period in winter while increasing steadily in summer. The content of total FFAs increased in both winter and summer, and polyunsaturated fatty acids (PUFAs) decreased significantly in summer. The characteristic compound in winter samples is hexanal, nonanal, and (E)‐2‐octenal, which may mainly come from the degradation of FAAs, while the characteristic compound in winter samples is hexanal, nonanal, and (E)‐2‐nonenal, which may mainly be derived from the oxidation of FFAs. This study extends our knowledge on flavor from traditional cured meat products at different processing stages in different seasons and could be useful for the standardization of the traditional and regional meat products.

## Graphical abstract

“Dao Ban Xiang” is a famous traditional Chinese dry‐cured meat product. This study aimed to comparatively analyze the difference in the volatile flavor information of “Dao Ban Xiang” produced in winter and summer. This study extends our knowledge on flavors from a traditional cured meat products at different processing stages in different seasons and could be useful for the standardization of the traditional and regional meat products.

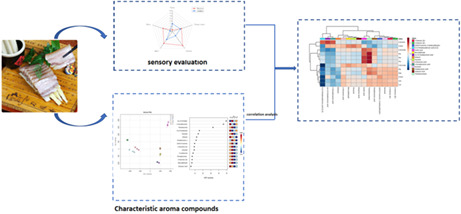

**Practical Application**

This study determined the key stages of DBX's characteristic flavor substances and flavor formation, which has a certain guiding significance for the subsequent industrial production and flavors the regulation of DBX.

## 1 INTRODUCTION

**FIGURE 1:**
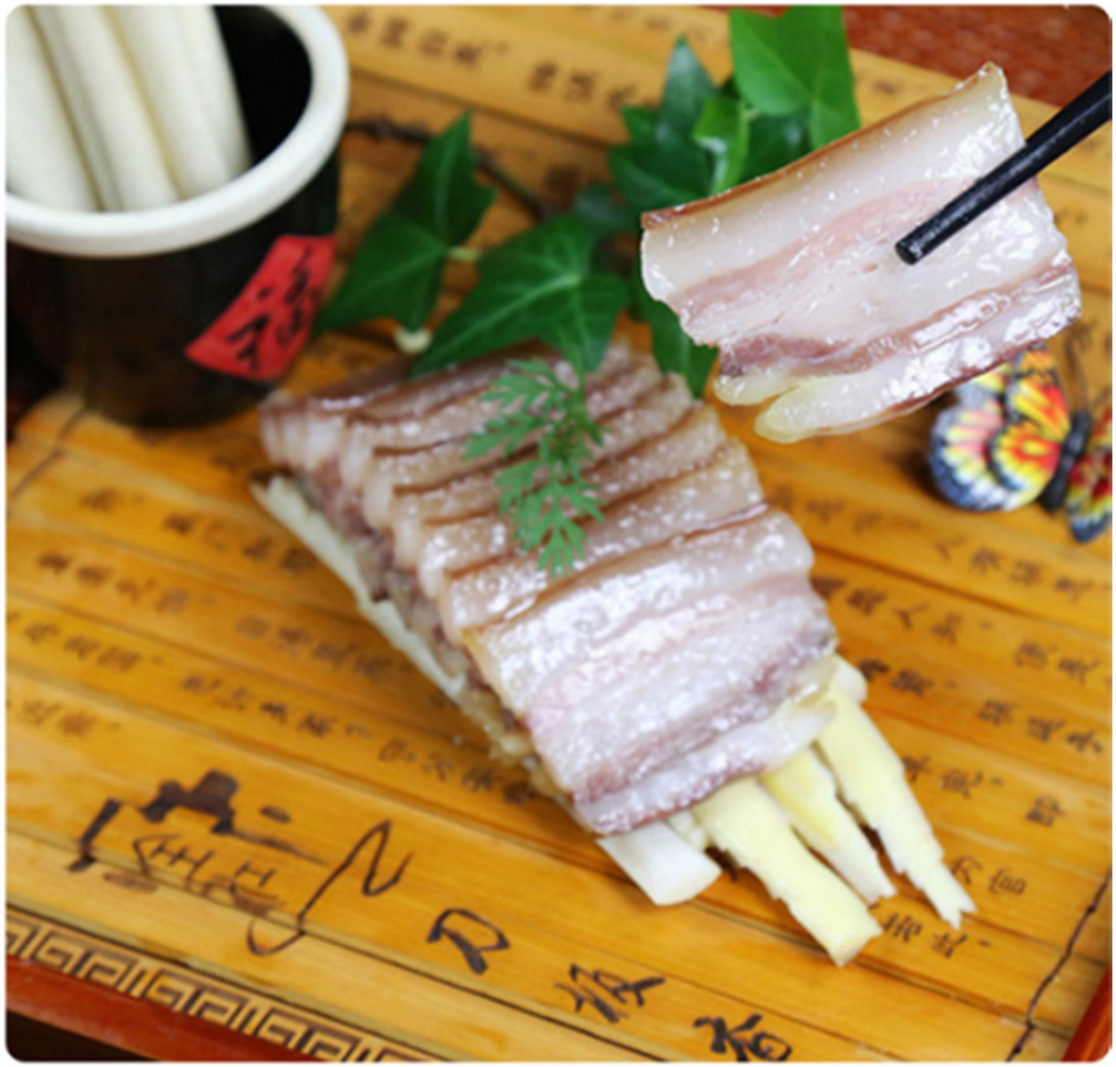
Dao Ban Xiang of Huizhou

The practice of curing meats for human consumption has been around for 1000 of years. And cured meat products are popular because of their desirable flavor profiles and convenient storage. “Dao Ban Xiang” (DBX) (Figure 1) is a traditional cured and sun‐dried meat product in Huizhou area, including parts of Anhui Province and Zhejiang Province of China. It is one of the indispensable delicacies in Huizhou cuisine (one of the eight traditional Chinese cuisines). “Dao Ban Xiang” in Chinese means that when cooking this kind of meat product, the fragrance will be left on the knife board. The raw material of DBX is streaky pork of local native pig (Wannanhua pig), which has a high‐fat content and is suitable for the production of bacon. The main process includes pretreatment, curing (30 days), and drying/natural fermentation (30 days in winter and 10 days in summer). As described by Wang et al. (2022), it is similar to bacon with a unique flavor (e.g., tangy, delicate, meaty, and salty), but unlike bacon, DBX is not smoked. The processing method of DBX mainly relies on traditional manual workshop production. And the traditional fermentation process is easily influenced by the local natural environment. In different seasons, different temperatures, humidity, corresponding microorganisms, and even the raw materials themselves may affect the flavor of the final product (Castellari et al., 2010; Comi et al., 2005; Gonzalez‐Rivas et al., 2020).

The formation of the flavor of cured meat products is a complex process in which the most important pathways are precursor degradation, lipid oxidation, and Maillard reaction. In addition to these three classical pathways, the influence of microorganisms on flavor is also critical. The most important microorganisms responsible for product transformation during fermentation are lactic acid bacteria (mainly *Lactobacillus* spp.) and coagulase‐negative cocci (*Staphylococcus* and *Kocuria* spp.), which seem to have the capacity to survive during fermentation (Gul et al., 2016). Shi et al. analyzed the flavor components responsible for the Dahe black pig dry‐cured ham and revealed that the flavor compounds mainly stem from the oxidation of fatty and degradation of amino acids (Shi et al., 2019). Laura et al. identified 102 volatile compounds from dry‐cured “lacón.” The results indicated that volatile compounds presented an increasing trend in the processing process, and lipids were the most important precursors of flavor compounds (Laura Purrinos et al., 2011). Although previous studies have focused on the influences of raw materials, processing technology, temperature, and cycle on the flavor of dry‐cured meat products (Sakaguchi, 1996; Zhang et al., 2011), there is little information on the flavor differences between dry‐cured meat products produced in winter and summer, respectively. Traditionally, two batches of DBX are produced every year, in winter and summer, respectively. The flavors of products in winter and summer are different through sensory evaluation. However, it remains unclear which characteristic flavoring substances of DBX are different in the two seasons and which flavor compounds are the key contributors to the difference.

Hence, the present study mainly aimed to investigate the flavor divergence of DBX produced in winter and summer, and further explore which flavor compounds are the key contributors to the divergence. It is conducive to optimizing the process, better‐standardizing production, and keeping the flavor of products produced in winter and summer consistent.

## 2 MATERIALS AND METHODS

### 2.1 Sample preparation

DBX samples in winter and summer were purchased from Huangshan Quanjiang Ecological Agriculture Co., Ltd. (Huangshan, China). The DBX was processed in the company according to the following process: Raw meat shall be pickled with 5% salt and an appropriate amount of alcohol and Sichuan pepper for 30 days (4°C). After washing the salt, it shall be hung outdoor for drying (30 days in winter (29°C) and 10 days in summer (6°C)). Here, samples of four stages (raw piece, curing, drying, and ripening) were taken for further analysis in both winter (November 2019 to January 2020) and summer (June to July 2020), and every time point was analyzed in three replicates. All samples were stored at −20°C after vacuum packaging. Before analysis, samples were thawed for 12 h at 4°C.

### 2.2 Sensory evaluation

After thawing the samples in winter and summer, slice them to ensure uniform thickness and place them on different plates for cooking for 30 min. Sensory analysis was carried out according to the description of Sirtori et al. (2019), with some modifications. Composed of 10 well‐trained judges (5 males and 5 females), the descriptive analysis method was used to study the five sensory characteristics of DBX, namely color, tissue state, texture, smell, and taste. The scoring criteria are shown in Table 1. The sensory room is designed according to the international standard (ISO 8589:2007), with comfortable temperature and good ventilation. It must not have a persistent odor, and the volume of the sound must be limited. There is no adsorption on the surface of the sensory chamber, and the design is convenient, hygienic, and clean. The size of the space needs to ensure comfort. Samples were placed in odorless transparent plastic dishes and numbered with three random numbers. During the evaluation process, the evaluators rested for 5 min after evaluating each group of samples. Repeat this process five times for all samples.

**TABLE 1:** Sensory evaluation form

Scoring criteria	Color	Tissue state	Texture	Smell	Taste
8–10	The fat is transparent, the lean meat is pink, and the skin is cash yellow	The tissue is tight and the muscle filaments remain in their original state	Moderate hardness and freshness	It has a strong flavor of wax, light, and fat	No spicy, salty, fresh The taste is palatable and tasteless
6–8	The fat is transparent, the lean meat is red, and the skin is golden yellow	The tissue is compact and the muscle filaments remain in their original state	It is fresh, tender, and refreshing, with moderate hardness and good elasticity	The flavor of wax, light, and fat is strong	No Hara flavor, salty taste, and delicious taste No smell
4–6	The transparency of fat is general, the lean meat is light red, and the skin is dark red	The tissue tightness is general, and the muscle filament remains in its original state	Fresh, tender, and refreshing, with general elasticity and slightly higher hardness	The smell of wax, light, and fat is average	No Hara flavor, the salty flavor is heavy, the fresh flavor is light, and there is no smell
2–4	The transparency of fat is dark, the lean meat is dark red, and the skin is dark gray	The tightness of the tissue and the muscle filament remain in the original state are poor	It is fresh, tender, and refreshing, with poor elasticity and high hardness	The smell of wax, light, and fat is average	It is slightly spicy, salty, light, and slightly delicious Taste
0–2	The fat is light yellow, the lean meat is dark gray, and the skin is white	Loose structure and poor formability	The entrance is hard and inelastic	No wax, fragrance, and grease	It has a strong spicy flavor

### 2.3 pH, moisture content, TBARS values, and salt content

The pH was measured using a digital pH meter (Mettler Toledo FE28‐Meter, Shanghai, China) equipped with a penetration probe. Moisture was quantified according to the ISO recommended standards 1442:1997 (ISO, 1997). Lipid oxidation was assessed in triplicate by the 2‐thiobarbituric acid (TBARS) method of Witte et al. (1970). Thiobarbituric acid reactive substance values were calculated according to the formula:

$$\mathrm{TBA}\;\left(\mathrm{mg}/100\;g\right)=\left(A532–A600\right)/155\times 1/5\times 72.6\times 100,$$

A532 and A600 mean the light absorption value at 532 nm and 600 nm and expressed as mg MDA/kg sample. The salt content was determined using an ISM‐146 Na Complete Sodium Measurement System (Lazar Research Laboratories, Inc., Los Angeles, CA, USA), previously calibrated with potassium bromide, and each sample was measured three times.

### 2.4 Free amino acids

The DBX samples were placed in oven at 121°C for 5 h and then cooled to room temperature. After obtaining the dry sample, accurately weigh 0.1 g of the sample dry sample, add 4 ml of 4% sulfosalicylic acid solution, and then conduct ultrasonic extraction for 30 min, turn it upside down every 5 min, and leave it for 10 min after extraction. Then, the supernatants were subsequently transferred to a 2 ml centrifuge tube and centrifuged at 2000 × g at 4°C for 30 min. After centrifugation, transfer 1 ml supernatant with 1 ml syringe for 0.22 μm water filtration membrane, put it into the injection bottle, and measure it with an amino acid automatic analyzer (S‐433D; Sykam, Germany).

### 2.5 Free fatty acids

5 g of DBX sample was added into a 50 ml centrifuge tube and mixed with 25 ml of n‐Hexane. After ultrasonic treatment for 20 min, stand for 2 h and then centrifuge at 4°C and 1000 × g for 15 min. The supernatant was put in a rotary steaming bottle for rotary evaporation (60°C, 80 r/min). After that, 20 μl lipids, 2 ml n‐Hexane, and 400 μl KOH‐methanol solution (1 mol/L) were mixed thoroughly and oscillation for 5 min. Then, anhydrous sodium sulfate was added to the sample and shaken for 2 min. After standing for layering, take 1 ml of supernatant, filter the organic membrane, and inject it into the sample bottle for measurement.

Separation and quantification of fatty acid methyl esters (FAME) were carried out using a gas chromatograph, GC‐Agilent 7693 N (Agilent Technologies lnc) equipped with a flame ionization detector and an automatic sample injector, and using PEG‐20 M capillary column (DB‐WAX, 30 m length, 0.25 mm diameter, 0.25 μm film thickness; Agilent Technologies Inc.). Chromatographic conditions were as follows: The temperature of the sample inlet and detector is 250°C, and the flow rate of carrier gas He is 3 ml/min. The injection volume was 1 μl with a split ratio of 10:1. Column temperature program was set as follows: 60°C (1 min), 60°C–200°C at 50°C/min 200°C (1 min), 200°C–250°C at 3°C/min, and 250°C (3 min). The fatty acids were quantified using nonadecanoic acid methyl ester at 0.3 mg/ml, as an internal standard, which was added to the samples before fat extraction and methylation. Identification of fatty acids was performed by comparison of the retention times to those of known fatty acids and the results expressed as mg/100 g of fat.

### 2.6 Analysis of volatile compounds

Four gram of DBX sample was minced and weighed into a 20 ml headspace vial together with cyclohexanone standard (947 μg/ml). The conditions of solid‐phase microextraction were measured by the method of Lorenzo (2014) with slight modifications. The vial was heated in a 60°C water bath, then exposed to solid‐phase microextraction (SPME) (75 μm CAR/PDMS) fibers in the headspace of the sample for 30 min. The compounds absorbed by the fibers were identified and quantified by gas chromatographic analysis using MS detectors.

Analyses were performed using a GCMS‐QP2010 Plus (Shimadzu, Japan) gas chromatograph equipped with the mass selective detector. The compounds were separated using a DB‐WAX capillary column (J&W Scientific: 20 m × 0.18 mm id, 0.18 μm film thickness). The SPME fiber was desorbed and maintained in the injection port at 280°C for 2 min. The sample was injected in splitless mode. Helium was used as a carrier gas with a linear velocity of 51 cm/s. The temperature program was isothermal for 2 min at 40°C, raised to 100°C at 6°C/min, and then raised to 240°C at 8°C/min and held for 5 min: total run time was 34.5 min. The injector and detector temperatures were both set at 280°C. The mass spectra were obtained using a mass selective detector working in electronic impact at 70 eV and collecting data at the range m/z 30–550. Compounds were identified by comparing their mass spectra with those contained in the NIST (National Institute of Standards and Technology, Gaithersburg, USA) library and confirmed by linear retention index (LRI) (Li et al., 2022). LRI was calculated using a mixture of *n*‐alkanes (C7–C30) as follows:

$$\mathrm{LRI}=100\times \left(n+\frac{T_{x}-T_{n}}{T_{n+1}-T_{n.}}\right),$$

where *T*_*x*_, *T*_*n*_, and *T*_*n* + 1_ are the retention times of compound *x*, alkane *n*, and alkane *n* + 1, respectively (*T*_*n*_ < *T*_*x*_ < *T*_*n* + 1_).

### 2.7 Statistical analysis

All data were expressed as mean ± SD (standard deviation) of three independent experiments. Single‐factor analysis of variance (ANOVA) was performed using SPSS software package (SPSS 22.0, Chicago, Illinois, USA). The Duncan multiple range test was used to compare the mean value, and *p* < .05 was defined as a significant difference. MetaboAnalyst 5.0 is used to perform the principal component analysis (PCA) and partial least‐squares discriminant analysis (PLS‐DA), examine the difference of volatile components, and determine the differential marker compounds in winter and summer samples (Li et al., 2022). The thermogram was used to explore the volatile compounds in samples and their correlation with sensory properties. Corresponding graphs were drawn with OmicShare (http://www.omicshare.com) (Al‐Dalali et al., 2022).

## 3 RESULTS AND DISCUSSION

### 3.1 Sensory analysis

**FIGURE 2:**
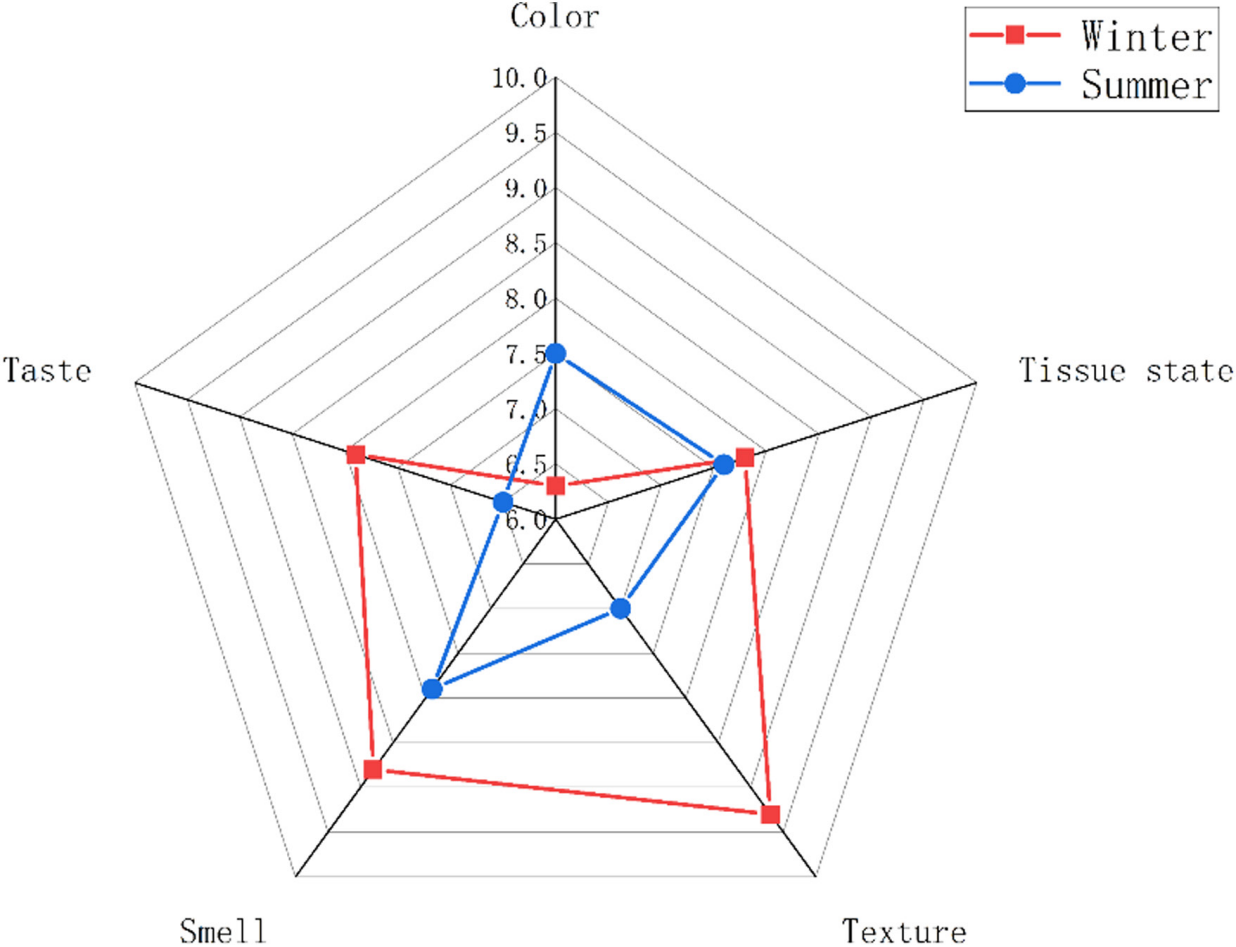
Sensory rating chart of DBX samples in winter and summer

The sensory characteristics of the sample of DBX in winter and summer were depicted in Figure 2. There was no significant difference in the score of tissue state between samples in winter and summer, while the score of overall taste, smell, and taste of winter samples was significantly higher than that of summer samples. Interestingly, the color score of summer samples is higher than that of winter samples, which may be due to the higher oxidation degree of summer samples compared with winter samples. The sensory results showed that compared with the samples in summer, the samples in winter had a stronger overall taste, smell, and more attractive taste characteristics.

### 3.2 Comparison of physicochemical properties

Physicochemical properties of DBX during different processing stages are summarized in Table 2. The pH differed significantly (*p* < .05) between DBXs from the two seasons. For the winter samples, a significant decrease was observed from raw piece to drying stage (from 6.35 to 5.96; *p* < .05) and the pH, then increased significantly (*p* < .05) to the end of the ripening process. A similar trend in pH changes was disclosed during the processing of other dry‐cured meat products (Lorenzo, 2014; Molinero et al., 2008). However, for the summer samples, the pH value showed significant (*p* < .05) fluctuations from raw to mature, and the pH value of the final stage is close to the winter samples. This is due to the increase in lactic acid caused by lactic acid bacteria during the product curing period and the decrease in pH value. In the air‐drying period, the environmental temperature of the sample in winter is low, and the lactic acid bacteria still have some activity. In the summer, the environmental temperature of the sample is too high, which leads to a decrease in the number of lactic acid bacteria, and the pH value will increase.

**TABLE 2:** Dynamic changes in physicochemical properties during the manufacture of “Dao Ban Xiang”

Chemical composition	Winter				Summer			
	Raw piece	Salting	Drying	Ripening	Raw piece	Salting	Drying	Ripening
Moisture (%)	37.12 ± 1.03^a^	35.60 ± 2.15^b^	29.96 ± 1.34^c^	23.31 ± 1.90^d^	41.29 ± 1.92^a^	39.23 ± 0.24^b^	28.11 ± 0.88^c^	24.92 ± 0.95^d^
PH	6.35 ± 0.01^a^	6.03 ± 0.06^c^	5.96 ± 0.03^c^	6.21 ± 0.04^b^	6.90 ± 0.01^a^	6.02 ± 0.06^d^	6.43 ± 0.03^b^	6.27 ± 0.05^c^
NaCl (g/100 g)	1.95 ± 0.31^d^	5.24 ± 0.20^a^	3.89 ± 0.33^b^	2.99 ± 0.39^c^	0.09 ± 0.01^d^	5.70 ± 0.72^a^	4.99 ± 0.82^b^	3.53 ± 0.31^c^
TBARS (mg MDA/kg)	0.47 ± 0.03^a^	0.80 ± 0.08^b^	0.69 ± 0.08^b^	0.72 ± 0.08^b^	0.33 ± 0.03^c^	0.42 ± 0.07^c^	0.83 ± 0.17^b^	1.54 ± 0.08^a^

*Note*: Mean values in the same row with different superscript letters differ significantly (*p* < .05), and results expressed as mean ± SE (*n* = 3).

The changes of water content in winter and summer were similar, and the average initial water contents of the raw pieces (37.12% and 41.29%) are significantly lower than the values found by other authors (Lorenzo, 2014; Molinero et al., 2008). The content decreases progressively during the processing period, being more pronounced during the last two steps. With the extension of drying time, the water evaporates and the water content decreases. The mean final values (23.31% and 24.92%) were significantly lower than those reported in other types of cured meats (Çakıcı et al., 2014; Lorenzo, 2014; Marra et al., 1999). It is mainly because it is placed outdoors when drying, which will have a certain air‐drying effect.

The sodium chloride content (expressed as g/100 g of dry matter) significantly (*p* < 0.05) increased during the salting as a result of salt diffusion, reaching maximum values after salting (5.24% and 5.70%). After the salting period, a slight drop was observed at the end of the process, eventually reaching the average value of 2.99 and 3.53 g/100 g of dry matter. The final average values were significantly lower than those reported by other authors (Garcia et al., 1997; Lorenzo, 2014; Molinero et al., 2008). Because the production cycle of knife plate incense is short, the amount of salt added is less, and there is a salt‐washing step in the processing process.

The oxidation degree of samples in winter and summer was measured by TBARS, which evaluates malonaldehyde formed in oxidation processes. The TBARS values in the raw pieces were 0.47 and 0.33 mg MDA/100 g in winter and summer, respectively, consistent with data reported by other authors in raw meat (Lorenzo, 2014; Lorenzo et al., 2008). TBARS values of winter increased significantly (*p* < 0.05) during the postsalting stage and showed a fluctuating trend after the salting stage. The increase in malonaldehyde contents during the postsalting stage in winter samples may be related to the prooxidant action of metallic ions present as impurities in the salt used in the curing process (Lorenzo, 2014). However, TBARS values of the summer sample increased significantly (*p* < .05) during the drying period from 0.42 to 1.54 mg malonaldehyde/100 g, and it may be due to more extensive oxidation caused by high temperatures in summer.

### 3.3 Comparison of free amino acids

The total free amino acids (FAAs) concentration (785.47 mg/100 g in winter and 1051.70 mg/100 g in summer) in DBX was significantly lower than that reported in other dry‐cured meat (Bermúdez et al., 2014; del Olmo et al., 2013; Pérez‐Palacios et al., 2011). The difference in FAAs levels in dry‐cured meat may arise from the effect of different processing conditions on enzyme activity and the different proteolytic activity of different pig breeds and slaughter age (Toldra, 1998).

The main FAAs in the raw pieces were glutamic, histidine, proline, and alanine in winter, and histidine, alanine, glycine, and lysine in summer. The amino acids alanine and histidine are also the main FAAs reported in raw materials of other dry‐cured meat (Bermúdez et al., 2014; Pérez‐Palacios et al., 2011; Virgili et al., 2007). However, at the end of processing, the FAAs with the highest concentration was histidine, followed by lysine, alanine, and leucine in winter. In summer samples, the dominant FAA was also histidine, followed by alanine, lysine, and arginine. This was similar to the FAAs observed in other types of dry‐cured meat (Bermúdez et al., 2014; del Olmo et al., 2013; Virgili et al., 2007).

His and Ala are the amino acids related to bitter and sweet tastes, respectively. Since their concentrations are above the threshold of sensory perception (20 mg/ml and 60 mg/ml, respectively) (Kato et al., 1989), histidine and alanine may contribute substantially to the taste properties of DBX. In addition, the concentration of glutamic in the DBX is also higher than in other types of dry‐cured meat (Bermúdez et al., 2014; del Olmo et al., 2013; Salazar et al., 2020).

The changes in different FAA content during the manufacture of DBX in winter and summer are shown in Table 3. The total average content of FAAs in winter decreased first and then increased, and the final content of FAAs dropped. However, the total average content of FAAs in summer increased first and then decreased, and the final content of FAAs raised, and this is in agreement with the results reported by other authors in dry‐cured meat (Salazar et al., 2015). FAAs except cysteine showed a significant decrease (*p* < .05) during the curing period for winter samples. However, in summer samples, FAAs except cysteine, histidine, and lysine showed a significant decrease (*p* < .05) only in the final stage of processing. No FAAs decrease in the curing period has been observed in other dry‐cured meat (Pérez‐Palacios et al., 2011; Salazar et al., 2015; Virgili et al., 2007). But previous studies also reported a decrease in FAAs during the final period of dry‐cured meat (Salazar et al., 2015; Toldra et al., 2000).

**TABLE 3:** Dynamic changes of FAA (mg/100 g of DM) during the manufacture of “Dao Ban Xiang”

FAA	Winter(mg/100 g of DM)				Summer(mg/100 g of DM)			
	Raw piece	Salting	Drying	Ripening	Raw piece	Salting	Drying	Ripening
Asp	9.48 ± 0.73^a^	7.27 ± 0.09^b^	5.49 ± 0.08^c^	9.58 ± 0.07^a^	28.02 ± 3.48^b^	30.35 ± 1.37^b^	19.46 ± 1.31^c^	45.07 ± 0.39^a^
Thr	15.4 ± 1.04^b^	9.19 ± 0.06^c^	15.32 ± 0.25^b^	36.7 ± 0.87^a^	33.19 ± 2.46^b^	25.18 ± 1.95^b^	159.84 ± 11.27^a^	24.95 ± 1.21^b^
Ser	20.89 ± 1.6^b^	11.15 ± 0.08^c^	20.04 ± 0.39^b^	45.17 ± 1.23^a^	49.3 ± 5.18^c^	74.03 ± 1.34^b^	23.63 ± 2.28^d^	84.41 ± 0.82^a^
Glu	240.65 ± 49.62^a^	7.83 ± 0.15^b^	13.72 ± 0.24^b^	36.38 ± 1.74^b^	15.98 ± 3.85^b^	5.69 ± 0.75^c^	22.3 ± 2.52^a^	12.6 ± 0.53^b^
Gly	24.59 ± 1.91^b^	11.94 ± 0.34^c^	13.79 ± 0.26^c^	33.61 ± 1.01^a^	46.56 ± 5.21^b^	33.05 ± 0.14^c^	54.69 ± 3.97^a^	34.34 ± 0.75^c^
Ala	54.73 ± 3.82^b^	27.73 ± 1.04^d^	37.84 ± 1.1^c^	69.06 ± 0.95^a^	103.2 ± 4.46^b^	111.28 ± 1.54^b^	125.33 ± 8.76^a^	105.64 ± 1.97^b^
Cys	5.13 ± 0.14^a^	5.3 ± 0.14^a^	3.6 ± 0.04^b^	1.13 ± 0.04^c^	4.5 ± 0.78^a^	1.75 ± 0.24^b^	1.17 ± 0.15^b^	1.18 ± 0.2^b^
Val	14.71 ± 1.03^c^	7.67 ± 0.15^d^	17.55 ± 0.25^b^	39.16 ± 1^a^	37.96 ± 3.88^c^	41.12 ± 0.51^c^	94.5 ± 5.72^a^	59.13 ± 0.15^b^
Met	9.57 ± 0.66^c^	4.42 ± 0.02^d^	10.94 ± 0.16^b^	25.03 ± 0.38^a^	12.98 ± 1.58^c^	21.24 ± 0.08^b^	54.83 ± 3.11^a^	19.66 ± 0.06^b^
Ile	12.89 ± 0.98^c^	7.53 ± 0.02^d^	20.25 ± 0.25^b^	38.6 ± 0.98^a^	22.86 ± 2.16^d^	43.84 ± 0.6^c^	86.46 ± 3.83^a^	55.11 ± 0.25^b^
Leu	23.99 ± 1.74^c^	13.71 ± 0.02^d^	32.69 ± 0.42^b^	62.24 ± 1.64^a^	35.8 ± 3.65^d^	67.18 ± 0.81^c^	136.47 ± 5.93^a^	88.18 ± 0.43^b^
Tyr	12.48 ± 0.95^c^	6.59 ± 0.2^d^	14.03 ± 0.13^b^	28.26 ± 0.64^a^	22.74 ± 2.28^d^	37.22 ± 0.53^c^	67.24 ± 2.95^a^	52.97 ± 0.03^b^
Phe	12.89 ± 0.98^c^	7.16 ± 0.07^d^	17.04 ± 0.41^b^	33.36 ± 0.57^a^	16.11 ± 1.69^d^	37.77 ± 0.71^c^	95.8 ± 3.47^a^	58.63 ± 0.4^b^
His	221.08 ± 5.81^a^	128.15 ± 2.64^c^	123.42 ± 3.1^c^	155.36 ± 3.47^b^	201 ± 9.05^b^	237.65 ± 3.17^a^	202.8 ± 12.87^b^	210.53 ± 5.28^b^
Lys	19.63 ± 1.77^c^	11.3 ± 0.25^d^	31.26 ± 0.76^b^	85.18 ± 2.5^a^	57.79 ± 5.21^c^	68.48 ± 0.3^c^	165.14 ± 11.15^a^	98.38 ± 0.98^b^
Arg	22.79 ± 1.45^b^	13.15 ± 0.67^c^	25.57 ± 0.72^b^	53.5 ± 1.79^a^	45.53 ± 4.53^c^	74.76 ± 0.38^b^	88.04 ± 4.8^a^	90.48 ± 1.15^a^
Pro	193.08 ± 39.61^a^	7.71 ± 0.15^b^	11.86 ± 0.68b	31.07 ± 1.14^b^	12.42 ± 2.9^b^	5.24 ± 0.82^c^	21.14 ± 1.29^a^	10.43 ± 0.44^b^
∑FAA	913.99 ± 110.50^a^	287.77 ± 5.13^b^	414.42 ± 7.21^b^	785.47 ± 23.99^a^	745.92 ± 55.42^d^	915.82 ± 8.69^c^	1418.84 ± 84.27^a^	1051.7 ± 7.42^b^

*Note*: Mean values in the same row with different superscript letters differ significantly (*p* < .05), and results expressed as mean ± SE (*n* = 3). ∑FAA refers to the total amount of all free amino acids in each stage.

The content of FAAs in DBX depends on the relationship between formation, degradation, and loss (Salazar et al., 2015; Toldra et al., 2000). The reduction in FAAs of winter samples during the curing period and summer samples at the end of the processing indicates that the degradation and loss of these compounds during this period are more active than their formation. The FAAs were lost to a certain extent due to the loss of water and salt‐washing process after curing; however, protease activity was higher in summer due to high temperature. For example, alanyl aminopeptidase is the enzyme responsible for the hydrolysis of a wide range of amino acids, and it is more active in summer (Toldra et al., 2000). At the end of processing, the activity of exopeptidase was lower due to the inhibition of salt, dehydration, and own enzymatic hydrolysis (Touraille & Girard, 1982). In addition, some amino acids undergo oxidative degradation during drying and maturation to form volatile compounds (Buscailhon et al., 1994). This further suggested that FAAs are an important source of the volatile compounds from dry‐cured meat products.

### 3.4 Comparison of free fatty acids

The changes in different free fatty acids (FFAs) contents during different processing stages of DBX in winter and summer were shown in Table 4. The total FFAs increased significantly in both winter and summer (*p* < .05) s. It was reported that the content of FFAs increased during processing in other dry‐cured meat products, for example, dry‐cured foal “cecina” (Lorenzo, 2014), dry‐cured loins (Guerrero et al., 1999), and dry‐cured sausages (Navarro et al., 1997). Since muscle enzyme systems play an important role in the generation of FFAs (Motilva et al., 1992), the increase in the amounts of FFA results in the action of lipolytic enzymes (Lorenzo, 2014).

**TABLE 4:** Dynamic changes of free fatty acids (mg/100 g fat) during the manufacture of “Dao Ban Xiang”

FFA	Winter(mg/100 mg fat)				Summer(mg/100 mg fat)			
	Raw piece	Salting	Drying	Ripening	Raw piece	Salting	Drying	Ripening
C6:0	3.94 ± 0.68^b^	3.33 ± 0.21^b^	1.82 ± 0.16^c^	2.19 ± 0.31^c^	4.76 ± 0.25^a^	1.78 ± 0.05^c^	1.87 ± 0.26^c^	3.22 ± 0.33^b^
C8:0	3.85 ± 0.19^a^	2.76 ± 0.36^b^	1.52 ± 0.18^c^	3.04 ± 0.11^b^	4.16 ± 0.34^a^	1.4 ± 0.13^c^	1.38 ± 0.08^c^	1.16 ± 0.02^c^
C10:0	4.06 ± 0.08^c^	4.99 ± 0.4^c^	8.43 ± 1.48^a^	5.15 ± 0.17^c^	5.15 ± 0.61^c^	6.68 ± 0.29^b^	6.6 ± 0.28^b^	7.64 ± 0.58^ab^
C11:0	1.19 ± 0.23^ab^	0.97 ± 0.18^ab^	0.97 ± 0.32^ab^	0.93 ± 0.03^bc^	1.38 ± 0.13^a^	0.65 ± 0.03^c^	0.71 ± 0.07^c^	0.66 ± 0.02^c^
C12:0	5.12 ± 0.2^c^	4.86 ± 0.51^c^	7.47 ± 1.3^a^	5.88 ± 0.24^bc^	5.47 ± 0.59^bc^	5.49 ± 0.37^bc^	5.92 ± 0.3^bc^	6.69 ± 0.11^ab^
C14:0	72.56 ± 0.36^c^	74.36 ± 4.45^bc^	110.18 ± 15.33^a^	107.77 ± 3.74^a^	76.77 ± 6.99bc	89.27 ± 4.97^b^	76.53 ± 2.75^bc^	103.52 ± 2.21^a^
C14:1	0.94 ± 0.06^c^	1.47 ± 0.13^b^	2.26 ± 0.17^a^	1.5 ± 0.02^b^	1 ± 0.09c	1.15 ± 0.05^c^	1.38 ± 0.13^b^	1.61 ± 0.03^b^
C15:0	2.09 ± 0.11^bcd^	2.14 ± 0.2^bc^	2.31 ± 0.28^ab^	1.7 ± 0.19^de^	2.59 ± 0.25^a^	1.8 ± 0.08^cde^	1.63 ± 0.16 ^e^	2.27 ± 0.19^ab^
C15:1	0.72 ± 0.09^bc^	0.65 ± 0.02^bc^	0.81 ± 0.11^ab^	0.59 ± 0.04^c^	0.7 ± 0.05^bc^	0.81 ± 0.09^ab^	0.69 ± 0.07^bc^	0.97 ± 0.15^a^
C16:0	1930.64 ± 130.62^b^	2245.42 ± 316.93^b^	2206.45 ± 192.1^b^	2941.11 ± 150.02^a^	2158.14 ± 235.41^b^	1947.72 ± 74.63^b^	2978.88 ± 81.81^a^	2956.69 ± 326.21^a^
C16:1	102.6 ± 1.21^c^	170.98 ± 8.26^a^	155.78 ± 15.84^ab^	164.13 ± 20.03^a^	123.02 ± 13.48^c^	130.97 ± 14.88^bc^	159.13 ± 7^ab^	172.79 ± 7.77^a^
C17:0	10.57 ± 0.09^b^	12.1 ± 1.24^ab^	15.67 ± 2.84^a^	9.44 ± 1.07^b^	14.79 ± 1.36^a^	9.68 ± 1.42^b^	9.26 ± 0.93^b^	12.81 ± 3.2^ab^
C17:1	11.29 ± 0.32^b^	15.65 ± 0.81^a^	12.3 ± 1.73^b^	10.26 ± 1.16^b^	14.65 ± 1.43^a^	11.52 ± 0.79^b^	10.9 ± 1.1^b^	11.78 ± 0.75^b^
C18:0	558.6 ± 2.18^d^	672.84 ± 34.49^cd^	884.16 ± 159.17^a^	867.03 ± 30.11^ab^	608.35 ± 19.28^cd^	959.75 ± 40.69^a^	739.88 ± 27.83^bc^	987.51 ± 57.43^a^
C18:1	2096.5 ± 170.4^c^	2123.2 ± 108.56^c^	2951.66 ± 368.25^ab^	3257.34 ± 133.01^a^	1946.46 ± 22.3^c^	3314.49 ± 140.57^a^	3172.29 ± 43.8^ab^	2804.05 ± 98.19^b^
C18:2	850.51 ± 4.61^a^	485.06 ± 24.67^d^	765.64 ± 31.9^b^	846.3 ± 29.09^a^	740.33 ± 23.52^b^	397.97 ± 30.45 ^e^	413.84 ± 13.46 ^e^	570.9 ± 11.89^c^
C18:3n6	1.69 ± 0.06^bc^	1.2 ± 0.02^d^	1.88 ± 0.31^ab^	1.65 ± 0.13^bc^	1.41 ± 0.04^cd^	2.06 ± 0.24^a^	1.37 ± 0.12^cd^	1.2 ± 0.12^d^
C18:3n3	53.65 ± 0.54^cd^	166.39 ± 9.64^b^	69.96 ± 5.16^c^	40.88 ± 1.33^de^	239.77 ± 25.37^a^	34.76 ± 1.52^de^	25.48 ± 2.71 ^e^	182.61 ± 9.71^b^
C20:0	10.39 ± 0.14^bc^	10.36 ± 1.02^bc^	12.12 ± 2.21^bc^	20.23 ± 0.7^a^	9.79 ± 1.02^c^	13.05 ± 1.95^b^	9.85 ± 1.06^c^	21.58 ± 0.41^a^
C20:1	44.06 ± 0.74^d^	41.02 ± 2.13^d^	65.64 ± 4.23^b^	63.29 ± 3.39^b^	2.14 ± 0.37 ^e^	52.61 ± 6.1^c^	55.49 ± 2.67^c^	76.19 ± 2.94^a^
C21:0	42.4 ± 10.85^a^	21.44 ± 2.18^c^	32.26 ± 2.06^b^	38.81 ± 1.39^ab^	1.22 ± 0.05^d^	22.28 ± 2.39^c^	15.37 ± 1.68^c^	40.75 ± 1.3^ab^
C22:0	5 ± 0.32^b^	3.34 ± 0.46^d^	4.67 ± 0.85^bc^	4.96 ± 0.6^b^	3.75 ± 0.37^cd^	4.56 ± 0.62^bc^	3.23 ± 0.39^d^	6.55 ± 0.04^a^
C20:2n6	11.53 ± 0.23^ab^	6.46 ± 0.36^c^	12.2 ± 2.33^a^	12.1 ± 1.2^a^	8.18 ± 1.07^bc^	12.4 ± 1.93^a^	8.76 ± 0.91^abc^	9.53 ± 2.57^abc^
C20:3n9	8.42 ± 0.6^d^	22.1 ± 1.32^c^	7.95 ± 0.97^d^	6.77 ± 0.2^de^	33.91 ± 1.05^b^	5.22 ± 0.55^ef^	4.41 ± 0.47^f^	40.8 ± 1.18^a^
C20:3n6	1.43 ± 0.05^d^	2.49 ± 0.14^bc^	2.49 ± 0.46^bc^	2.06 ± 0.22^cd^	3.23 ± 0.24^b^	2.13 ± 0.37^cd^	1.89 ± 0.28^cd^	4.72 ± 0.76^a^
C22:1n9	–	–	–	–	–	–	–	1.05 ± 0.25
C23:0	–	1.23 ± 0.1^b^	–	1.77 ± 0.23^b^	1.51 ± 0.14^b^	1.37 ± 0.24^b^	1.06 ± 0.17^b^	3.15 ± 0.07^a^
C22:2	–	1.1 ± 0.01^a^	1.07 ± 0.24^a^	0.73 ± 0.09^b^	0.59 ± 0.04^b^	0.81 ± 0.08^b^	0.22 ± 0.31^c^	0.97 ± 0.34^c^
C20:5	0.58 ± 0.04 ^e^	0.85 ± 0.1^cde^	1.38 ± 0.26^b^	0.93 ± 0.11^cde^	0.71 ± 0.06^de^	1.23 ± 0.2^bc^	1 ± 0.1^bcd^	2.9 ± 0.31^a^
C24:0	4.02 ± 0.28^ef^	7.39 ± 0.43^c^	6.09 ± 1.17^cd^	3.44 ± 0.3^f^	9.15 ± 0.92^b^	5.33 ± 0.83^de^	4.28 ± 0.49^ef^	15.64 ± 0.64^a^
∑SFA	2554.41 ± 13.11^c^	3067.54 ± 297.65^bc^	3448.92 ± 307.08^b^	4013.44 ± 168.86^a^	2905.59 ± 239.65^bc^	3070.57 ± 106.43^bc^	3856.22 ± 112.79^a^	3949.93 ± 86.28^a^
∑MUFA	2374.85 ± 11.09^c^	2352.97 ± 109.97^c^	3422.11 ± 159.25^ab^	3456.76 ± 141.18^a^	2087.97 ± 14.74^c^	3511.57 ± 136.90^a^	3346.85 ± 37.44^ab^	3090.84 ± 109.83^b^
∑PUFA	925.41 ± 3.97^b^	685.29 ± 29.87 ^e^	846.18 ± 20.90^cd^	911.41 ± 31.11bc	1028.13 ± 40.80^a^	456.57 ± 33.91^f^	456.98 ± 13.42^f^	806.29 ± 19.12^d^
∑FFA	5838.36 ± 32.58^d^	6105.8 ± 159.27^d^	7345.15 ± 543.91^c^	8421.97 ± 316.41^a^	6021.69 ± 273.29^d^	7038.71 ± 264.4^c^	7660.05 ± 153.22^bc^	8051.91 ± 323.74^ab^

*Note*: Mean values in the same row with different superscript letters differ significantly (*p* < .05), and results expressed as mean ± SE (*n* = 3). ∑ refers to the sum of substances.

The amounts of saturated fatty acids (SFAs) and monounsaturated fatty acids (MUFAs) of the FFAs fraction significantly increased (*p* < .05) during the production of DBX in winter and summer. But, the content of polyunsaturated fatty acids (PUFAs) significantly decreased (*p* < .05) during processing. The result was consistent with previous reports (Martın et al., 1999). The decrease of PUFAs in FFAs during ham processing may be due to their high susceptibility to oxidation, while SFAs and MUFAs remained stable or increased. The contents of SFAs and MUFAs are similar in winter and summer, while the content of PUFAs in summer is significantly lower (*p* < .05) than that in winter. This phenomenon may be partially explained by the high temperature in summer, and it can also be reflected by the TBA value during the drying period of samples in summer.

The content of all FFAs increased except linoleic acid (C18:3n6) during processing. Linoleic acid (C18:3n6) content increased first and then decreased in both winter and summer. The reduction range of linoleic acid (C18:3n6) in summer samples is much larger than that in winter, and the final content decreased significantly in summer. Linoleic acid (C18:3n6) will undergo an oxidation reaction during the air‐drying period, and the higher temperature and oxidation degree in summer will lead to a much lower content of linoleic acid (C18:3n6). The content of linoleic acid (C18:3n6) in other cured meat products has declined during processing (Martın et al., 1999; Yang et al., 2005), but the opposite trend has also been reported (Yang et al., 2017).

The automatic oxidation of linoleic acid will lead to the formation of many volatile compounds. The oxidative decomposition of the unsaturated part of linoleic acid involves a free radical chain reaction, which is carried out through the formation of hydroperoxide. Hydrogen peroxide does not have its own flavor. Therefore, their subsequent decomposition will produce carbonyl compounds, alcohols, ketones, furans, epoxides, and hydrocarbons (Shahidi & Oh, 2020). During the processing of DBX, palmitic (C16:0), stearic (C18:0), and oleic acid (C18:2) are the main free fatty acids. A significant increase in these FAAs contents was observed during processing (*p* < .05). And the drying stage is the dominant stage, which is consistent with the previous studies (Lorenzo, 2014; Zhang et al., 2011).

### 3.5 Comparison of volatile compounds

A total of 70 volatile compounds, classified into 6 chemical families (alcohols, aldehydes, ketones, esters, acids, and other compounds), were detected during the production process of DBX. The compound content is shown in Table 5. The total amount of volatile compounds increased significantly during the process in both winter and summer, from 1638.97 to 14815.91 and 358.43 to 655.33 mg/100 g, respectively (*p* < .05). But, the total amount of volatile compounds is not always rising during the processing process in summer, showing a trend of rising first and then falling. At the end of processing, the most abundant volatile compounds were acids and aldehydes in winter and summer, respectively. This result is consistent with other reports on dry‐cured meat (Purrinos et al., 2011; Sánchez‐Peña et al., 2005). The main source of flavor substances in cured meat products is lipid oxidation, which mainly generates aldehydes and acids.

**TABLE 5:** Dynamic changes of volatile compounds (mg/100 of meat) during the manufacture of “Dao Ban Xiang”

Chemical class	RT	Odor threshold	Winter								Summer							
			Raw piece		Salting		Drying		Dring‐ripening		Raw piece		Salting		Drying		Dring‐ripening	
			Content	OAV	Content	OAV	Content	OAV	Content	OAV	Content	OAV	Content	OAV	Content	OAV	Content	OAV
Alcohols																		
Ethanol	2.715	95,000	35.23 ± 1.59^c^	<0.01	17.29 ± 0.92^c^	<0.01	326.37 ± 20.50^b^	<0.01	162.61 ± 12.46^bc^	<0.01	0.00 ± 0.00^c^	–	1487.20 ± 270.23^a^	0.02	69.04 ± 0.77^c^	<0.01	41.80 ± 2.91^c^	<0.01
1‐Pentanol	8.86	400	3.62 ± 0.19^cde^	0.01	7.39 ± 3.54^c^	0.02	20.03 ± 3.13^b^	0.05	28.68 ± 0.43^a^	0.07	5.75 ± 0.34^cd^	0.01	20.35 ± 1.66^b^	0.05	3.12 ± 0.15^de^	0.01	1.44 ± 0.23 ^e^	<0.01
2‐Heptenal, (Z)‐	11.15	ND	5.39 ± 0.43^d^	–	24.45 ± 15.64^bc^	–	33.90 ± 2.63^ab^	–	41.23 ± 0.88^a^	–	7.69 ± 1.33^d^	–	7.00 ± 0.20^d^	–	13.64 ± 1.18^cd^	–	4.20 ± 0.14^d^	–
1‐Hexanol	11.25	5000	5.27 ± 0.16^d^	<0.01	4.21 ± 0.85^d^	<0.01	13.82 ± 0.74^c^	<0.01	36.72 ± 0.53^a^	0.01	2.94 ± 0.52 ^e^	<0.01	18.39 ± 0.87^b^	<0.01	0.00 ± 0.00^f^	–	0.98 ± 0.06^f^	<0.01
1‐Octen‐3‐ol	13.36	15.853	3.72 ± 0.06^c^	0.23	14.35 ± 0.62^c^	0.91	0.00 ± 0.00^c^	–	241.27 ± 29.99^a^	15.22	0.00 ± 0.00^c^	–	44.68 ± 0.98^b^	2.82	0.00 ± 0.00^c^	–	12.39 ± 1.82^c^	0.78
1‐Heptanol	13.505	4250	2.85 ± 1.55^d^	<0.01	13.26 ± 1.82^c^	<0.01	15.11 ± 1.80^c^	<0.01	92.01 ± 3.51^a^	0.02	3.54 ± 0.27^d^	<0.01	26.30 ± 0.76^b^	0.01	3.82 ± 0.77^d^	<0.01	0.67 ± 0.05^d^	<0.01
1‐Tetradecanol	14.51	ND	1.86 ± 0.10^c^	–	9.33 ± 1.89^b^	–	0.00 ± 0.00^d^	–	58.31 ± 0.93^a^	–	0.00 ± 0.00^d^	–	0.00 ± 0.00^d^	–	0.00 ± 0.00^d^	–	1.65 ± 0.15^cd^	–
1‐Octanol	15.47	1900	2.31 ± 0.44^c^	<0.01	8.83 ± 1.26^c^	<0.01	6.18 ± 0.73^c^	<0.01	123.03 ± 24.18^a^	0.06	5.27 ± 0.30^c^	<0.01	28.53 ± 1.74^b^	0.02	2.92 ± 0.59^c^	<0.01	1.27 ± 0.09^c^	<0.01
2‐Octenal, (E)‐	16.47	ND	3.28 ± 0.07^d^	–	54.89 ± 5.27^c^	–	76.62 ± 1.47^b^	–	329.55 ± 13.58^a^	–	5.75 ± 0.05^d^	–	71.07 ± 4.44^b^	–	8.10 ± 0.58^d^	–	3.02 ± 1.76^d^	–
Phenylethyl Alcohol	21.015	1400	1.92 ± 0.28^d^	<0.01	5.34 ± 0.11^c^	<0.01	10.42 ± 0.63^b^	0.01	13.56 ± 0.46^a^	0.01	0.00 ± 0.00 ^e^	–	0.00 ± 0.00 ^e^	–	0.47 ± 0.02 ^e^	<0.01	0.52 ± 0.00 ^e^	<0.01
1‐Dodecanol	21.775	160	1.43 ± 0.26 ^e^	0.01	9.60 ± 1.16^d^	0.06	38.22 ± 0.80^b^	0.24	44.08 ± 0.80^a^	0.28	1.32 ± 0.07 ^e^	0.01	19.41 ± 3.45^c^	0.12	1.39 ± 0.30 ^e^	0.01	1.90 ± 0.28 ^e^	0.01
n‐Heptadecanol‐1	28.785	ND	0.00 ± 0.00^b^	–	14.33 ± 2.76^a^	–	0.00 ± 0.00^b^	–	0.00 ± 0.00^b^	–	1.51 ± 0.05^b^	–	0.00 ± 0.00^b^	–	0.00 ± 0.00^b^	–	0.83 ± 0.05^b^	–
Aldehydes																		
Hexanal	5.03	45	5.00 ± 0.14 ^e^	0.11	38.36 ± 0.67^de^	0.85	225.40 ± 28.42^c^	5.01	230.78 ± 23.11^c^	5.13	60.08 ± 0.78^d^	1.34	766.30 ± 22.65^a^	17.03	564.17 ± 20.43^b^	12.54	49.27 ± 3.67^d^	1.09
Heptanal	7.48	28	6.72 ± 0.11 ^e^	0.24	57.92 ± 0.13^d^	2.07	116.15 ± 3.83^b^	4.15	153.44 ± 3.17^a^	5.48	0.00 ± 0.00^f^	–	90.57 ± 3.44^c^	3.23	8.56 ± 1.55 ^e^	0.31	8.31 ± 0.18 ^e^	0.3
Octanal	9.64	32	11.61 ± 0.51^c^	0.36	41.08 ± 2.20^b^	1.28	53.70 ± 3.09^b^	1.68	242.19 ± 27.62^a^	7.57	8.42 ± 0.31^c^	0.26	55.06 ± 7.77^b^	1.72	35.94 ± 1.52^b^	1.12	2.63 ± 0.05^c^	0.08
Nonanal	12.06	27.1	13.11 ± 1.67 ^e^	0.48	63.83 ± 6.81^d^	2.36	225.1 ± 14.60^c^	8.31	760.47 ± 38.95^a^	28.06	14.37 ± 0.50 ^e^	0.53	282.26 ± 10.50^b^	10.42	24.97 ± 1.62 ^e^	0.92	8.28 ± 0.16 ^e^	0.31
2‐Octenal, (E)‐	12.83	65	3.94 ± 0.95^d^	0.13	54.89 ± 5.27^c^	1.83	76.62 ± 1.47^b^	2.55	329.55 ± 13.58^a^	10.99	4.48 ± 0.92^d^	0.15	57.74 ± 8.16^c^	1.92	8.43 ± 0.56^d^	0.28	5.36 ± 0.11^d^	0.18
Decanal	14.265	3500	2.05 ± 0.43^d^	0.03	7.72 ± 1.07^c^	0.12	0.00 ± 0.00^d^	–	62.03 ± 2.73^a^	0.95	0.00 ± 0.00^d^	–	19.32 ± 0.37^b^	0.3	0.00 ± 0.00^d^	–	0.00 ± 0.00^d^	–
Benzaldehyde	14.67	14	3.48 ± 0.14^d^	<0.01	7.27 ± 0.34^cd^	<0.01	132.08 ± 27.53^b^	0.04	155.75 ± 3.32^a^	0.04	3.56 ± 0.30^d^	<0.01	27.17 ± 0.63^c^	0.01	7.93 ± 0.06^cd^	<0.01	4.63 ± 0.65^d^	<0.01
2‐Nonenal, (E)‐	14.965	154	4.95 ± 0.68 ^e^	0.35	35.61 ± 2.23^d^	2.54	73.23 ± 9.38^c^	5.23	241.14 ± 27.63^a^	17.22	6.37 ± 0.17 ^e^	0.46	58.76 ± 3.08^c^	4.2	9.91 ± 1.35 ^e^	0.71	131.75 ± 4.57^b^	9.41
2,4‐Heptadienal, (E,E)‐	15.63	30	1.30 ± 0.08 ^e^	0.01	15.73 ± 2.74^c^	0.1	21.11 ± 0.69^b^	0.14	41.42 ± 5.28^a^	0.27	0.00 ± 0.00 ^e^	–	3.31 ± 0.46^de^	0.02	2.42 ± 0.37 ^e^	0.02	7.81 ± 0.21^d^	0.05
Dodecanal	17.99	630	0.00 ± 0.00^d^	–	0.00 ± 0.00^d^	–	36.05 ± 0.75^b^	0.06	51.91 ± 1.88^a^	0.08	0.00 ± 0.00^d^	–	5.27 ± 0.59^c^	0.01	1.18 ± 0.12^d^	<0.01	0.00 ± 0.00^d^	–
2‐Undecenal	18.645	14	8.88 ± 0.82^d^	0.63	85.47 ± 57.06^b^	6.11	184.23 ± 10.46^b^	13.16	378.67 ± 23.36^a^	27.05	9.05 ± 0.56^d^	0.65	16.73 ± 1.03^d^	1.19	23.15 ± 3.05^d^	1.65	5.29 ± 0.92^d^	0.38
2‐Decenal, (E)‐	18.68	170	7.16 ± 1.06^d^	0.04	87.77 ± 9.56^c^	0.52	158.26 ± 25.67^b^	0.93	352.54 ± 30.83^a^	2.07	0.00 ± 0.00^d^	–	6.92 ± 0.16^d^	0.04	22.66 ± 1.73^d^	0.13	4.45 ± 0.35^d^	0.03
2,4‐Decadienal, (E,E)‐	18.84	100	3.74 ± 0.24^d^	0.04	87.40 ± 0.29^b^	0.87	105.77 ± 37.62^ab^	1.06	130.50 ± 4.81^a^	1.3	0.00 ± 0.00^d^	–	34.82 ± 3.55^c^	0.35	2.97 ± 0.21^d^	0.03	3.52 ± 0.15^d^	0.04
2,4‐Decadienal	19.54	3	0.00 ± 0.00^d^	–	252.99 ± 22.50^a^	84.33	257.09 ± 16.59^a^	85.7	186.28 ± 12.62^b^	62.09	0.00 ± 0.00^d^	–	0.00 ± 0.00^d^	–	0.00 ± 0.00^d^	–	4.84 ± 0.79^d^	1.61
Hexadecanal	23.95	9100	2.71 ± 0.11^d^	<0.01	19.16 ± 1.47^c^	<0.01	39.63 ± 2.70^b^	<0.01	129.46 ± 7.13^a^	0.01	0.00 ± 0.00^d^	–	0.00 ± 0.00^d^	–	4.18 ± 0.14^d^	<0.01	0.00 ± 0.00^d^	–
4‐Heptenal	24.01	42	2.60 ± 0.08^d^	0.06	5.83 ± 0.67^bc^	0.14	6.14 ± 0.26^ab^	0.15	5.34 ± 0.32^c^	0.13	0.00 ± 0.00 ^e^	–	6.59 ± 0.25^a^	0.16	0.57 ± 0.15 ^e^	0.01	0.00 ± 0.00 ^e^	–
9‐Octadecenal, (Z)‐	28.67	ND	1.51 ± 0.07 ^e^	–	31.39 ± 4.34^c^	–	45.03 ± 0.82^b^	–	118.52 ± 12.01^a^	–	0.00 ± 0.00 ^e^	–	17.14 ± 1.72^d^	–	0.00 ± 0.00 ^e^	–	0.88 ± 0.06 ^e^	–
Ketones																		
3‐Octen‐2‐one	12.35	0.67	0.00 ± 0.00^d^	–	0.00 ± 0.00^d^	–	21.25 ± 0.54^b^	31.72	81.87 ± 3.10^a^	122.2	0.00 ± 0.00^d^	–	0.00 ± 0.00^d^	–	0.00 ± 0.00^d^	–	0.00 ± 0.00^d^	–
3,5‐Octadien‐2‐one	17.24	15	0.00 ± 0.00^d^	–	7.72 ± 1.59^b^	0.51	17.26 ± 1.16^a^	1.15	0.00 ± 0.00^d^	–	0.00 ± 0.00^d^	–	0.00 ± 0.00^d^	–	1.41 ± 0.19^d^	0.09	3.08 ± 0.55^c^	0.21
1‐Hexanone, 1‐phenyl‐	22.365	ND	1.12 ± 0.05^d^	–	7.31 ± 1.45^cd^	–	9.56 ± 0.36^cd^	–	23.43 ± 17.66^ab^	–	1.69 ± 0.13^d^	–	18.97 ± 1.57^abc^	–	4.15 ± 0.17^d^	–	32.01 ± 1.37^a^	–
4H‐Pyran‐4‐one, 2‐ethyl‐3‐hydroxy‐	22.47	350,000	535.56 ± 93.26^a^	<0.01	0.00 ± 0.00^c^	–	0.00 ± 0.00^c^	–	492.75 ± 5.18^a^	<0.01	0.00 ± 0.00^c^	–	525.89 ± 36.01^a^	<0.01	131.26 ± 23.07^b^	<0.01	136.48 ± 12.33^b^	<0.01
3‐Decen‐2‐one	22.48	ND	0.00 ± 0.00^d^	–	0.00 ± 0.00^d^	–	0.00 ± 0.00^d^	–	42.43 ± 1.04^a^	–	0.00 ± 0.00^d^	–	0.00 ± 0.00^d^	–	0.00 ± 0.00^d^	–	0.00 ± 0.00^d^	–
2‐Pentadecanone	24.45	ND	0.00 ± 0.00^d^	–	27.38 ± 1.92^a^	–	0.00 ± 0.00^d^	–	0.00 ± 0.00^d^	–	2.31 ± 0.07^b^	–	0.00 ± 0.00^d^	–	0.00 ± 0.00^d^	–	0.00 ± 0.00^d^	–
2‐Nonadecanone	25.14	ND	0.96 ± 0.26^d^	–	9.22 ± 0.69^c^	–	54.91 ± 5.49^a^	–	16.37 ± 1.10^b^	–	0.00 ± 0.00^d^	–	9.63 ± 0.45^c^	–	0.00 ± 0.00^d^	–	0.00 ± 0.00^d^	–
2(3H)‐Furanone, 5‐dodecyldihydro‐	32.165	ND	5.99 ± 0.45^d^	–	0.00 ± 0.00^d^	–	122.36 ± 2.10^b^	–	473.13 ± 13.70^a^	–	0.00 ± 0.00^d^	–	29.27 ± 0.90^b^	–	2.15 ± 0.12^d^	–	0.00 ± 0.00^d^	–
Ester compounds																		
Octanoic acid, ethyl ester	13	920	3.77 ± 0.49^d^	<0.01	0.00 ± 0.00^d^	–	0.00 ± 0.00^d^	–	138.56 ± 17.09^a^	0.15	0.00 ± 0.00^d^	–	23.90 ± 1.64^a^	0.03	0.00 ± 0.00^d^	–	4.72 ± 0.2^d^	0.01
Decanoic acid, ethyl ester	16.825	230	1.95 ± 0.34^d^	0.01	0.00 ± 0.00 ^e^	–	0.00 ± 0.00 ^e^	–	69.00 ± 0.81^a^	0.3	0.00 ± 0.00 ^e^	–	33.01 ± 0.86^b^	0.14	1.53 ± 0.15^d^	0.01	8.09 ± 1.23^c^	0.04
Ethyl 9‐decenoate	17.68	ND	0.66 ± 0.11^bc^	–	0.00 ± 0.00^c^	–	0.00 ± 0.00^c^	–	0.00 ± 0.00^c^	–	0.00 ± 0.00^c^	–	9.07 ± 1.18^a^	–	0.00 ± 0.00^c^	–	1.25 ± 0.21^b^	–
n‐Caproic acid vinyl ester	18.98	ND	0.00 ± 0.00^d^	–	13.86 ± 2.11^b^	–	0.00 ± 0.00^d^	–	0.00 ± 0.00^d^	–	0.00 ± 0.00^d^	–	40.13 ± 1.67^a^	–	4.36 ± 0.20^c^	–	0.00 ± 0.00^d^	–
Octadecanoic acid, ethyl ester	22.855	ND	0.55 ± 0.07^c^	–	0.00 ± 0.00^c^	–	0.00 ± 0.00^c^	–	24.85 ± 1.68^a^	–	0.00 ± 0.00^c^	–	5.36 ± 0.26^b^	–	0.00 ± 0.00^c^	–	0.00 ± 0.00^c^	–
Hexadecanoic acid, methyl ester	24.96	ND	0.63 ± 0.08^d^	–	14.35 ± 1.24^b^	–	39.43 ± 3.52^a^	–	13.83 ± 1.89^b^	–	0.77 ± 0.05^d^	–	0.00 ± 0.00^d^	–	0.00 ± 0.00^d^	–	0.00 ± 0.00^d^	–
Hexadecanoic acid, ethyl ester	25.395	20,000	5.13 ± 0.31^d^	<0.01	0.00 ± 0.00 ^e^	–	75.7 ± 1.67^a^	<0.01	64.40 ± 3.64^b^	<0.01	0.00 ± 0.00 ^e^	–	0.00 ± 0.00 ^e^	–	0.00 ± 0.00	–	9.89 ± 0.40^c^	<0.01
Ethyl 9‐hexadecenoate	25.715	ND	1.10 ± 0.30^c^	–	0.00 ± 0.00^c^	–	0.00 ± 0.00^c^	–	42.82 ± 1.19^a^	–	0.00 ± 0.00^c^	–	0.00 ± 0.00^c^	–	0.00 ± 0.00^c^	–	11.89 ± 0.49^b^	–
Ethyl Oleate	27.965	ND	6.22 ± 0.44^b^	–	25.15 ± 0.64^a^	–	0.00 ± 0.00^d^	–	0.00 ± 0.00^d^	–	0.00 ± 0.00^d^	–	0.00 ± 0.00^d^	–	0.00 ± 0.00^d^	–	2.56 ± 0.39^b^	–
Acids																		
Acetic acid	14.69	5000	0.00 ± 0.00^c^	–	65.33 ± 2.30^b^	0.01	151.82 ± 2.02^a^	0.03	167.02 ± 26.71^a^	0.03	12.60 ± 0.43^c^	<0.01	0.00 ± 0.00^c^	–	21.25 ± 0.75^c^	<0.01	15.85 ± 0.64^c^	<0.01
Propanoic acid	15.07	21,900	2.05 ± 0.16^de^	<0.01	3.19 ± 0.25^d^	<0.01	23.39 ± 1.02^b^	<0.01	106.23 ± 3.40^a^	<0.01	0.00 ± 0.00 ^e^	–	14.56 ± 0.42^c^	<0.01	1.62 ± 0.09^de^	<0.01	0.00 ± 0.00 ^e^	–
Butanoic acid	16.66	240	1.20 ± 0.22^d^	<0.01	12.49 ± 0.84^c^	0.05	22.39 ± 0.89^b^	0.09	81.53 ± 6.44^a^	0.34	1.73 ± 0.11^d^	0.01	13.17 ± 2.99^c^	0.05	0.98 ± 0.12^d^	<0.01	0.64 ± 0.04^d^	<0.01
Pentanoic acid	18.445	1100	1.04 ± 0.12 ^e^	<0.01	6.09 ± 0.54^d^	0.01	17.85 ± 2.54^b^	0.02	10.62 ± 0.32^c^	0.01	3.18 ± 0.40^de^	<0.01	31.64 ± 3.13^a^	0.03	2.95 ± 0.48^de^	<0.01	2.12 ± 0.09 ^e^	<0.01
Hexanoic acid	20.05	30,000	7.92 ± 0.25^d^	<0.01	66.43 ± 7.09^c^	<0.01	127.81 ± 7.53^b^	<0.01	146.11 ± 4.98^b^	<0.01	14.12 ± 2.42^d^	<0.01	351.70 ± 28.17^a^	0.01	31.92 ± 4.49^d^	<0.01	7.04 ± 0.59^d^	<0.01
Octanoic Acid	22.985	30,000	3.42 ± 0.99^d^	<0.01	23.11 ± 1.91^b^	<0.01	126.93 ± 8.30^a^	<0.01	126.43 ± 10.03^a^	<0.01	15.48 ± 2.38^bc^	<0.01	120.80 ± 5.22^a^	<0.01	11.37 ± 0.26^cd^	<0.01	7.53 ± 0.92^cd^	<0.01
Heptanoic acid	23.49	700	0.00 ± 0.00 ^e^	<0.01	16.23 ± 0.65^b^	<0.01	6.86 ± 0.18^c^	<0.01	0.00 ± 0.00 ^e^	<0.01	0.00 ± 0.00 ^e^	<0.01	46.15 ± 2.69^a^	<0.01	4.40 ± 1.18^d^	<0.01	4.36 ± 0.38^d^	<0.01
Nonanoic acid	24.33	16,000	3.54 ± 0.70 ^e^	<0.01	13.22 ± 2.02^d^	<0.01	30.05 ± 3.29^c^	<0.01	85.48 ± 7.25^b^	<0.01	15.95 ± 2.48^d^	<0.01	127.26 ± 8.25^a^	<0.01	13.98 ± 2.01^d^	<0.01	3.66 ± 0.52 ^e^	<0.01
Dodecanoic acid, 3‐hydroxy‐	24.56	ND	0.61 ± 0.08^c^	–	0.00 ± 0.00^d^	–	0.00 ± 0.00^d^	–	50.09 ± 0.32^a^	–	0.00 ± 0.00^d^	–	9.66 ± 0.47^b^	–	0.00 ± 0.00^d^	–	0.00 ± 0.00^d^	–
n‐Decanoic acid	25.61	1000	5.65 ± 0.90^d^	0.01	85.23 ± 1.84^c^	0.09	214.92 ± 21.32^b^	0.21	410.21 ± 28.33^a^	0.41	14.34 ± 0.40^d^	0.01	60.72 ± 5.40^c^	0.06	16.03 ± 1.09^d^	0.02	26.75 ± 1.99^d^	0.03
9‐Decenoic acid	26.33	ND	1.25 ± 0.44 ^e^	–	20.30 ± 0.38^c^	–	37.95 ± 1.24^b^	–	69.26 ± 3.64^a^	–	0.00 ± 0.00 ^e^	–	0.00 ± 0.00 ^e^	–	22.97 ± 1.77^c^	–	5.65 ± 0.15^d^	–
Pentadecanoic acid	31.595	100,000	9.38 ± 6.59^d^	<0.01	88.17 ± 9.55^c^	<0.01	183.76 ± 10.54^b^	<0.01	288.07 ± 3.79^a^	<0.01	0.00 ± 0.00^d^	–	8.26 ± 0.21^d^	<0.01	0.00 ± 0.00^d^	–	0.00 ± 0.00^d^	–
n‐Hexadecanoic acid	33.155	3200	188.80 ± 62.54^d^	0.06	2242.64 ± 272.90^b^	0.7	2907.41 ± 196.12^a^	0.91	2188.83 ± 53.56^b^	0.68	130.33 ± 2.93^d^	0.04	557.43 ± 29.33^c^	0.17	55.15 ± 1.31^d^	0.02	50.25 ± 1.58^d^	0.02
Octadecanoic acid	37.635	200,000	238.02 ± 150.15^b^	<0.01	69.29 ± 3.03^c^	<0.01	224.31 ± 17.93^b^	<0.01	770.22 ± 42.12^a^	<0.01	0.00 ± 0.00^c^	–	25.78 ± 2.69^c^	<0.01	26.60 ± 2.56^c^	<0.01	0.00 ± 0.00^c^	–
9,12‐Octadecadienoic acid (Z,Z)‐	40.16	ND	169.43 ± 114.24^c^	–	463.18 ± 39.44^b^	–	927.88 ± 80.15^a^	–	124.32 ± 1.09^c^	–	0.00 ± 0.00^d^	–	0.00 ± 0.00^d^	–	8.03 ± 0.11^d^	–	0.00 ± 0.00^d^	–
cis‐13‐Octadecenoic acid	40.76	ND	291.29 ± 8.37^c^	–	2137.52 ± 104.83^b^	–	0.00 ± 0.00^d^	–	2243.96 ± 32.35^a^	–	0.00 ± 0.00^d^	–	0.00 ± 0.00^d^	–	0.00 ± 0.00^d^	–	0.00 ± 0.00^d^	–
Other compounds																		
Furan, 2‐pentyl‐	8.715	58	0.00 ± 0.00^d^	–	15.03 ± 0.85^b^	0.26	0.00 ± 0.00^d^	–	29.09 ± 0.86^a^	0.5	0.00 ± 0.00^d^	–	9.44 ± 1.48^c^	0.16	0.00 ± 0.00^d^	–	0.00 ± 0.00^d^	–
2,4‐Nonadienal, (E,E)‐	17.845	ND	1.54 ± 0.32^d^	–	0.00 ± 0.00^d^	–	52.58 ± 1.76^b^	–	119.61 ± 17.52^a^	–	0.00 ± 0.00^d^	–	18.90 ± 1.08^c^	–	2.60 ± 0.47^d^	–	2.13 ± 0.23^d^	–
Hexadecane	17.85	30,000	2.46 ± 0.12^d^	<0.01	10.99 ± 1.64^a^	<0.01	0.00 ± 0.00 ^e^	<0.01	8.76 ± 0.55^b^	<0.01	0.00 ± 0.00 ^e^	<0.01	6.76 ± 0.20^c^	<0.01	0.70 ± 0.04 ^e^	<0.01	2.09 ± 0.08^d^	<0.01
Formamide, N,N‐dibutyl‐	19.05	ND	1.53 ± 0.63^c^	–	15.99 ± 1.28^c^	–	62.28 ± 1.98^b^	–	115.05 ± 19.19^a^	–	0.00 ± 0.00^c^	–	0.00 ± 0.00^c^	–	0.31 ± 0.01^c^	–	0.00 ± 0.00^c^	–
Benzene, 1‐methoxy‐4‐(1‐propenyl)‐	19.765	ND	0.55 ± 0.1^d^	–	5.05 ± 0.48^b^	–	2.81 ± 0.44^c^	–	4.32 ± 0.06^b^	–	2.99 ± 0.21^c^	–	24.03 ± 1.03^a^	–	3.26 ± 0.43^c^	–	1.46 ± 0.04^d^	–
Octadecane	21.245	500	0.00 ± 0.00^c^	–	14.26 ± 1.12^a^	0.03	0.00 ± 0.00^c^	–	0.00 ± 0.00^c^	–	1.78 ± 0.06^b^	<0.01	0.00 ± 0.00^c^	–	1.36 ± 0.1^b^	<0.01	0.00 ± 0.00^c^	–
Heneicosane	26.6	ND	0.00 ± 0.00^d^	–	21.99 ± 1.38^a^	–	0.00 ± 0.00^d^	–	0.00 ± 0.00^d^	–	1.05 ± 0.04^d^	–	6.64 ± 0.26^c^	–	0.54 ± 0.04^d^	–	7.92 ± 0.06^b^	–
Tetratriacontane	27.8	ND	0.00 ± 0.00^d^	–	65.93 ± 5.13^c^	–	535.1 ± 0.69^b^	–	1446.18 ± 4.41^a^	–	0.00 ± 0.00^d^	–	0.00 ± 0.00^d^	–	0.00 ± 0.00^d^	–	0.58 ± 0.05^d^	–

*Note*: Mean values in the same row with different superscript letters differ significantly (*p* < .05), and results expressed as mean ± SE (*n* = 3).

**FIGURE 3:**
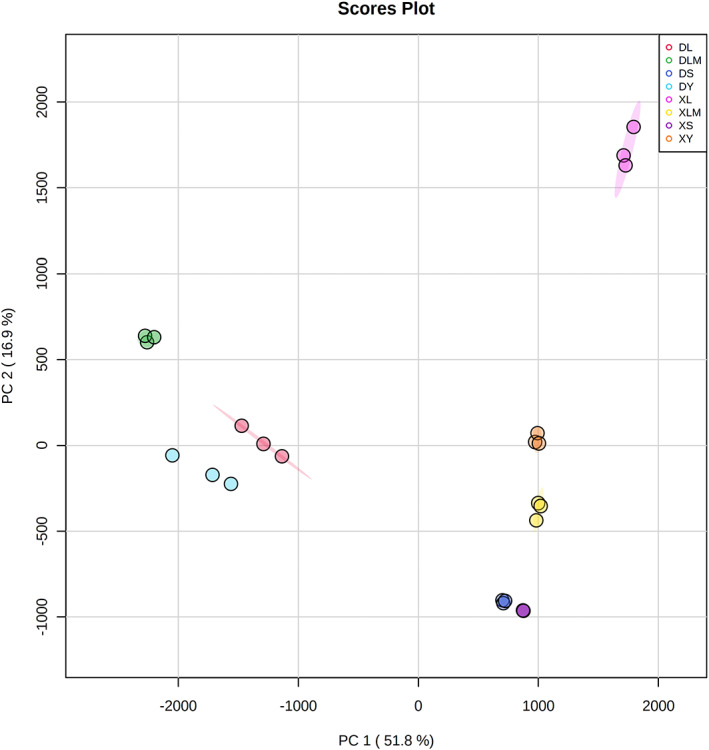
Plot of the principal component analysis on the flavor features of the “Dao Ban Xiang” during different ripening stages in winter and summer. Each dot represents an individual sample. DS, raw piece in winter; DY, salting in winter; DL, drying in winter; DLM, ripening in winter; XS, raw piece in summer; XY, salting in summer; XL, drying in summer; XLM, ripening in summer.

To better understand the relationship among processing stages, the principal component analysis (PCA) was performed (Figure 3). The data dots changed greatly in the curing and ripening stages for winter samples; however, the summer samples changed greatly in the curing stage and little in the dry‐ripening stage, and the data dots of raw pieces samples in winter and summer overlapped on PC1 and PC2. The results indicated that the volatile flavor compounds of raw meat samples were similar in winter and summer. The main formation stage of flavor in winter was the salting and ripening stage, while the main flavor formation stage in summer was the salting stage. Therefore, the formation of DBX flavor might be controlled by the curing and ripening stages. The curing period is a process of selective survival of microorganisms and enzymes in meat. During this period, microorganisms and enzymes that play a major role in the formation of DBX can be screened, so this period is the key stage to determine the characteristic flavor of DBX.

**FIGURE 4:**
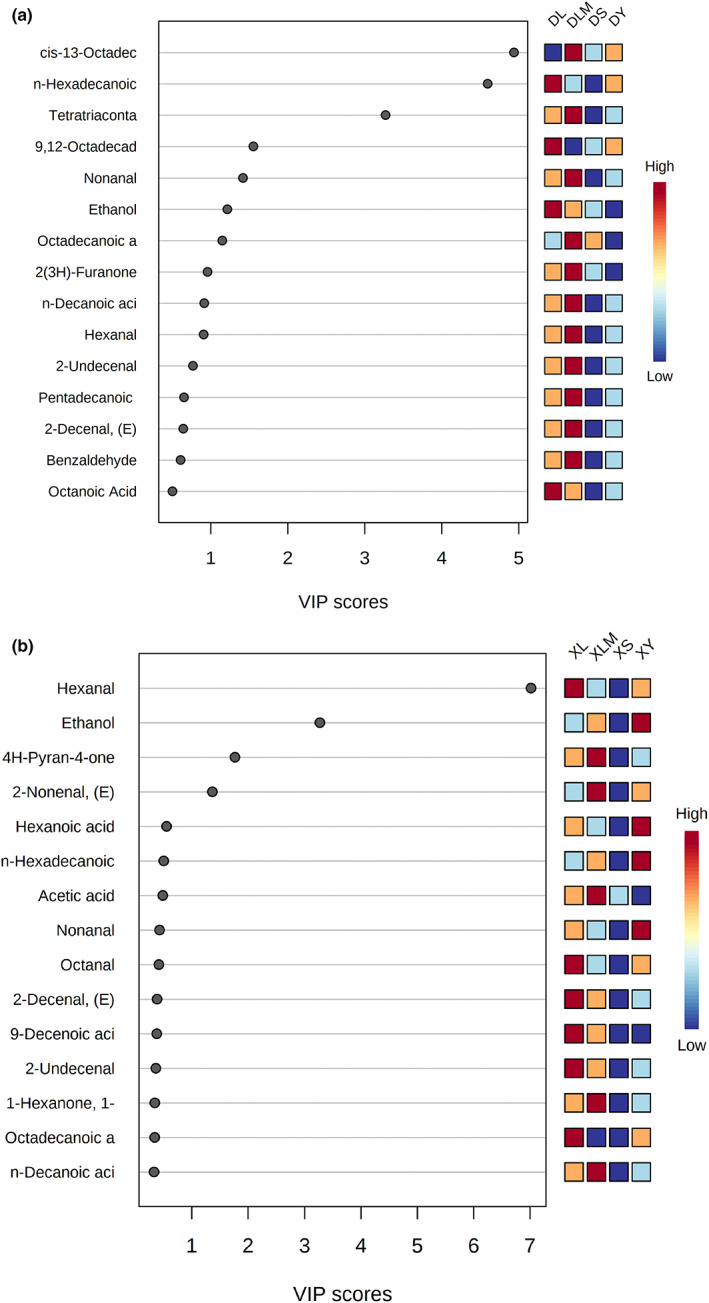
VIP diagram of volatile compounds from samples in winter (a) and summer (b)

To further examine the contribution of volatile compounds to product flavor, we introduce odor activity value (OAV) as a screening criterion (Feng et al., 2018; Qian et al., 2021). Generally, compounds (with OAV > 1) are considered as an important contributor to product flavor. As shown in Figure 4, the farther the compounds were located from the origin in the loading plot, the more important they were for the differentiation pattern. PCA loading (PLS‐DA and OPLS‐DA) was performed to analyze compounds with drastic changes in the processing process and to determine the key flavor compounds of DBX. According to the S‐plot, we selected the candidate compounds with VIP values larger than 1 and then considered them as key flavor compounds when the corresponding OAV value is greater than 1. The key flavor compounds in both winter and summer are aldehydes, including hexanal, nonanal, and (E)‐2‐octenal in winter, while hexanal, nonanal, and (E)‐2‐nonenal in summer. Hexanal and nonanal are also the odor active compounds of dry‐cured meat products such as Xuanwei ham, Istrian dry‐cured ham, and dry‐cured foal loin (Lorenzo & Carballo, 2015; Marusic et al., 2011; Wang et al., 2018), and (E)‐2‐octenal, (E)‐2‐nonenal were found in Jinhua ham (Liu et al., 2009). Aldehydes are secondary products of lipid oxidation. Due to their low odor threshold, they play an important role in the flavor of dry‐cured meat products. Hexanal and nonanal are mainly derived from the oxidative decomposition of linoleic acid (C18:3n6) and may also partly come from the Strecker degradation of some amino acids (Lorenzo & Carballo, 2015; Ruiz et al., 2002).

In addition to the characteristic compound aldehydes, other compounds such as acids, alcohols, and ketones also changed sharply during processing. These substances may also play an important role in the formation of flavor during processing, so it is important to analyze the sources of these substances for understanding the formation of flavor substances.

### 3.6 Correlation analysis of volatile compounds with free amino acids and fatty acids

The formation of the flavor of dry‐cured meat products is mainly related to the oxidation of fat, the hydrolysis of protein, and the degradation of free amino acids (Marusic et al., 2011). The decomposition of lipids and the oxidative degradation of free fatty acids and amino acids lead to the formation of numerous volatile compounds, such as aldehydes, ketones, alcohols, and short‐chain fatty acids. These compounds play an important role in the formation of unique flavors of dry‐cured meat products (Domínguez et al., 2019). To analyze the relationship between free amino acids, free fatty acids, and the formation of volatile compounds, we analyzed the correlation between the main free amino acids, free fatty acids, and volatile compounds in the processing.

**FIGURE 5:**
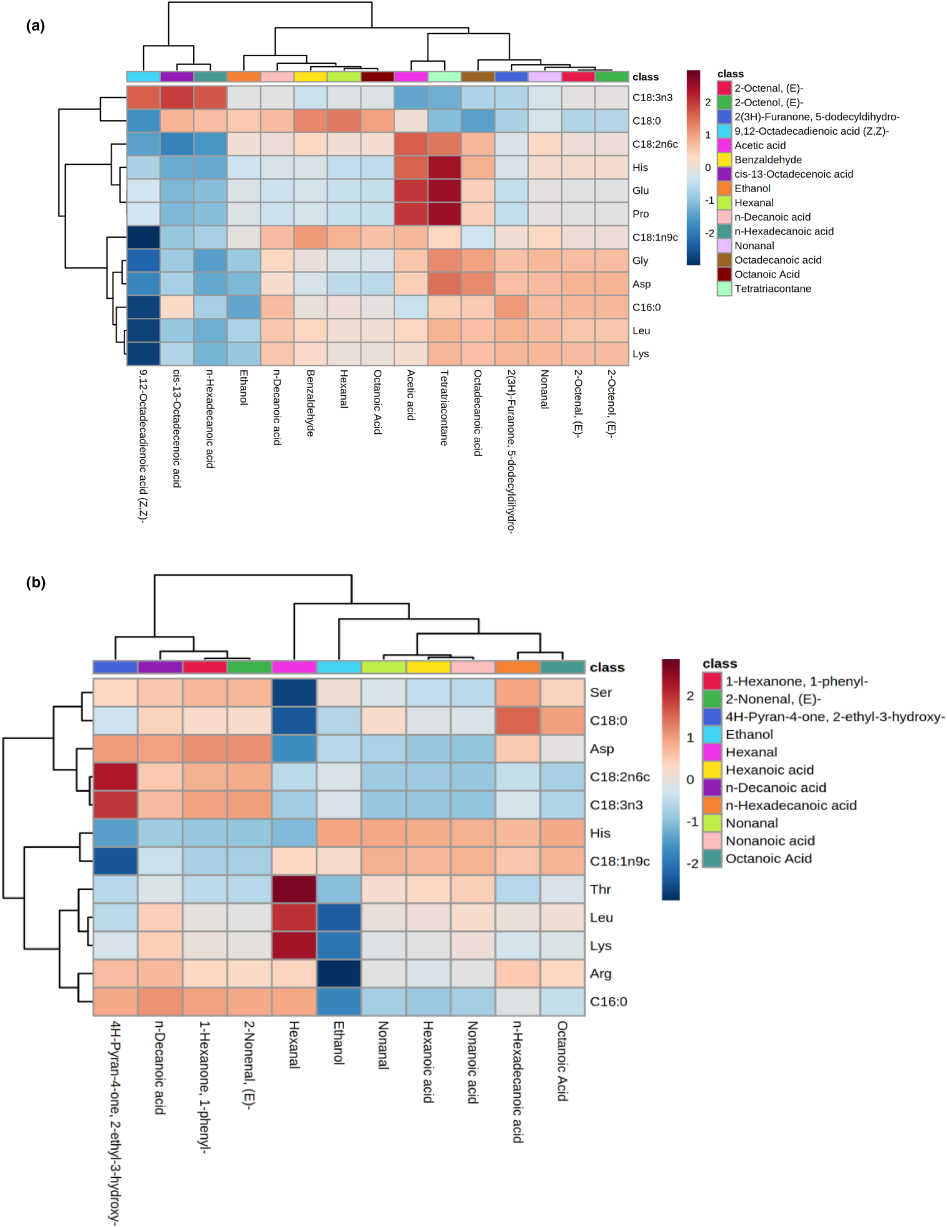
Correlation heatmap of free amino acids, free fatty acids, and volatile compounds from samples in winter (a) and summer (b)

As depicted in Figure 5a, Glu, His, Pro, and α‐Linolenic acid (C18:3n3) is negatively correlated with most volatile compounds in winter and might be the main source of volatile compounds in winter samples. Glu, His, and Pro are highly negatively correlated with cis‐13‐octadecenoic acid and palmitic acid, and α‐Linolenic acid (C18:3n3) is highly negatively correlated with acetic acid. Dry‐cured meat products will produce a large number of organic acids and fatty acids, mainly from the deamination of amino acids and the decomposition of free fatty acids. Acid compounds have an important contribution to the aroma of dry‐cured meat products, with weak pungent, aldehyde, and tallow aroma, and they can also produce other aroma compounds through side reactions (Chen et al., 2017, 2021). However, as shown in Figure 5b, α‐Linolenic acid (C18:3n3) and palmitic acid (C16:0) are negatively correlated with most volatile compounds in summer, which might be the main sources of volatile compounds in summer. Palmitic acid (C16:0) is highly negatively correlated with aldehydes and acids compounds. Aldehydes are the secondary products of lipid oxidation, mainly from the oxidation of free fatty acids. With a low‐odor threshold, aldehydes play an important role in the flavor of dry‐cured meat products. For example, the main flavor hexanal in this experiment has the vanilla smell, which mainly comes from the lipid oxidation of linoleic acid (C16:0). Other saturated aldehydes may come from the autooxidation of unsaturated fatty acids such as linoleic acid (C18:3n6) and linolenic acid (C18:3n3).

## 4 CONCLUSIONS

In this study, the volatile compounds in winter and summer samples were identified by GC–MS. The characteristic flavor compounds in winter samples were identified as hexanal, nonanal, and (E)‐2‐octenal, as well as hexanal, nonanal, and (E)‐2‐nonenal in summer samples. Correlation analysis shows that the volatile compounds may mainly come from Glu, His, Pro and α‐Linolenic acid, and α‐Linoleic acid and palmitic acid. By comparing and analyzing the flavor differences of DBX produced in winter and summer, this study expanded our understanding of the flavor of dry‐cured meat products and provided a basis for the standardization of traditional and regional meat products.

## CONFLICT OF INTEREST

The author declares no conflicts of interest.

## Data Availability

Data sharing is not applicable to this article as no new data were created or analyzed in this study.
